# Supplementary material for: Phylogenetic divergence of GABAB receptor signaling in neocortical networks over adult life
Source: Nat Commun. 2025 May 6;16:4194. doi: 10.1038/s41467-025-59262-8 (PMC12056048; doi:10.1038/s41467-025-59262-8)
Supplement: Supplementary file 1 — Supplementary Information [file 41467_2025_59262_MOESM1_ESM.pdf]

Wilson et al., *Phylogenetic divergence of GABAB receptor signalling in neocortical networks over adult life.*

Age	Sex	Hemi-sphere	Region	Seizure History	LEV	WC	LFP
25-30	F	Left	Temporal	Yes	Yes	3	1
30-35	M	Left	Frontal	No	No	2	
30-35	M	Left	Frontal	No	No	3	1
30-35	F	Left	Temporal	Yes	Yes	4	
30-35	F	Left	Frontal	Yes	Yes	3	
30-35	F	Left	Frontal	No	No	2	
35-40	F	Right	Frontal	No	No	3	
35-40	F	Left	Frontal	Yes	Yes	1	
35-40	F	Left	Frontal	Yes	Yes	4	1
40-45	F	Right	Frontal	No	No	2	1
40-45	M	Right	Frontal	Yes	Yes		
45-50	M	Right	Parietal	Yes	Yes	4	1
45-50	F	Right	Frontal	No	Yes	5	
45-50	F	Left	Frontal	No	Yes	3	1
45-50	M	Left	Temporal	No		1	
45-50	M	Left	Frontal	No	No	7	
45-50	M	Right	Frontal	Yes	Yes	6	
50-55	M	Right	Frontal	No	No		1
50-55	F	Left	Frontal	Yes	Yes		1
50-55	M	Right	Temporal	No	No	2	
50-55	M	Left	Frontal	No	Yes		1
50-55	M	Left	Parietal	No	No	2	
55-60	M	Right	Temporal	Yes	Yes	17	
55-60	M	Right	Frontal	No	Yes	3	1
55-60	M	Left	Parietal	Yes	Yes	3	
55-60	M	Right	Parietal	No	No		1
60-65	F	Right	Frontal	No	No	3	
60-65	F	Left	Occipital	Yes	Yes	4	
60-65	F	Left	Parietal	Yes	Yes	2	
60-65	M	Left	Frontal	Yes	Yes	7	
60-65	M	Right	Frontal	No	No	2	
60-65	M	Left	Temporal	Yes	Yes	2	
60-65	F	Left	Frontal	No	No		1
60-65	M	Right	Temporal	No		2	
60-65	M	Left	Frontal	No	No		1
65-70	M	Right	Frontal	No		1	
65-70	M	Left	Frontal	No	No	5	
65-70	M	Left	Temporal	No	No	7	1
65-70	M	Right	Frontal	No	No	3	
65-70	M	Left	Temporal	No	Yes	3	
65-70	M	Left	Parietal	Yes	Yes	3	1
65-70	F	Right	Parietal	Yes	Yes	1	
65-70	F	Left	Temporal	No		1	
70-75	M	Bifrontal	Frontal	Yes	Yes		1
70-75	M	Left	Frontal	No	No	3	

70-75	M	Right	Frontal	No	No	2	
70-75	M	Right	Parietal	No	No	3	1
70-75	F	Right	Temporal	No	No	3	
70-75	M	Right	Temporal	No	No	4	
75-80	M	Left	Frontal	No	No	2	
75-80	F	Right	Temporal	Yes	Yes	4	1
75-80	M	Right	Parietal	No	No		1
75-80	M	Left	Temporal	No	No	4	

Supplementary Table 1: Summary of human case details. A summary of the key clinical features of the patients consented for the current study, according to age. Listing the sex at birth, brain region, hemisphere, reason for surgery, history of seizures, prescription of levetiracetam (LEV), and number of replicates included in this study from whole-cell (WC) and local field potential (LFP) recordings.

Electrophysiological Property	1M rat L2/3 26 cells, 14 rats	1M rat L5 25 cells,13 rats	6-8 M rat L2/3 35 cells, 20 rats	6-8 M rat L5 31 cells, 20 rats	12-14M rat L2/3 13 cells, 5 rats	12-14M rat L5 11 cells, 5 rats	P (layer)	P (age)	P (interact)
Membrane potential (mV)	-77.3 ± 5.2	-66.1 ± 4.9	-74.6 ± 6.8	-66.6 ± 5.3	-71.0 ± 9.2	-67.0 ± 3.7	**$3.1x10^{-11}$**	0.318	**0.030**
Input resistance (MΩ)	48.4 ± 16.3	82.5 ± 43.4	79.8 ± 57.4	81.5 ± 52.6	77.7 ± 58.3	63.3 ± 24.5	0.536	0.132	**0.005**
Membrane time-constant (ms)	11.1 ± 2.3	12.8 ± 3.5	11.5 ± 5.3	16.0 ± 6.0	11.0 ± 4.1	14.8 ± 3.6	**$2.0x10^{-5}$**	0.715	0.125
Capacitance (pF)	248.5 ± 76.3	184.2 ± 91.3	168.8 ± 66.4	257.7 ± 140.5	175.4 ± 54.1	256.2 ± 84.4	0.092	0.663	**$4.5x10^{-6}$**
Rheobase (pA)	409.5 ± 151.3	220.0 ± 95.7	342.9 ± 133.5	196.8 ± 98.3	276.9 ± 142.3	154.5 ± 68.8	**$3.8x10^{-11}$**	0.040	0.439
Voltage threshold (mV)	-39.4 ± 3.2	-39.3 ± 6.8	-37.1 ± 3.4	-44.4 ± 5.6	-43.9 ± 6.7	-49.2 ± 7.9	**$4.5x10^{-5}$**	**0.003**	**0.0004**
AP amplitude (mV)	126.6 ± 5.7	107.3 ± 5.7	118.8 ± 11.3	105.7 ± 9.4	114.5 ± 14.1	108.4 ± 7.1	**$7.5x10^{-14}$**	0.089	**0.006**
AP 20-80% rise-time (ms)	0.13 ± 0.02	0.14 ± 0.04	0.15 ± 0.03	0.13 ± 0.03	0.14 ± 0.04	0.12 ± 0.02	0.215	0.091	**0.015**
AP half-height width (ms)	0.82 ± 0.15	0.65 ± 0.12	0.79 ± 0.14	0.65 ± 0.11	0.68 ± 0.07	0.60 ± 0.08	**$1.5x10^{-10}$**	0.143	0.492
AP maximum rise ($mV.ms^{-1}$)	503.6 ± 98.0	420.6 ± 96.9	411.0 ± 111.0	423.1 ± 120.5	490.3 ± 101.2	566.6 ± 126.6	0.830	**0.024**	**0.0007**
AP maximum decay ($mV.ms^{-1}$)	93.9 ± 16.9	122.3 ± 23.3	97.5 ± 15.2	137.0 ± 30.9	113.6 ± 13.1	147.8 ± 26.6	**$3.8x10^{-14}$**	**0.021**	0.322
FI Slope ($AP. pA^{-1}$)	0.013 ± 0.014	0.043 ± 0.019	0.022 ± 0.019	0.041 ± 0.016	0.040 ± 0.015	0.043 ± 0.009	**0.017**	**$2.9x10^{-9}$**	**0.0008**

Supplementary Table 2: Electrophysiological properties of rats 1-, 6-8-, and 12-14-month-old rat PCs. Key intrinsic physiological measurements made from L2/3 and L5 PCs. Data is shown as mean ± SD. Statistics shown as p-values from linear mixed effects models (LMM) comparing the effect of age, layer, and the interaction of both. Number of cells and rats contributing to each dataset are indicated.

Electrophysiological Property	Adult rat L2/3 48 cells, 25 rats	Adult rat L5 42 cells, 25 rats	Human L2/3 37 cell, 20 cases	Human L5 16 cell, 9 cases	P (spec.)	P (layer)	P (interact)
Membrane potential (mV)	-73.6 ± 7.6	-66.7 ± 4.9	-65.5 ± 5.9	-68.6 ± 5.6	0.387	**0.001**	**0.008**
Input resistance (MΩ)	79.3 ± 57.0	76.7 ± 47.3	120.0 ± 101.9	101.3 ± 61.2	0.799	0.822	0.740
Membrane time-constant (ms)	11.3 ± 5.0	15.7 ± 5.4	16.1 ± 7.9	16.7 ± 8.1	**0.007**	**0.002**	0.056
Capacitance (pF)	170.6 ± 62.8	257.3 ± 127.2	183.7 ± 86.6	218.6 ± 138.1	0.576	**0.002**	0.064
Rheobase (pA)	325.0 ± 137.6	185.7 ± 92.6	206.1 ± 136.3	193.8 ± 118.1	0.264	**0.002**	**0.006**
Voltage threshold (mV)	-39.0 ± 5.4	-45.6 ± 6.6	-41.2 ± 5.9	-42.2 ± 7.7	0.801	**0.0001**	**0.012**
AP amplitude (mV)	117.6 ± 12.1	106.4 ± 8.8	112.2 ± 10.5	105.1 ± 12.4	0.128	**5.0×10^{-7}**	0.221
AP 20-80% rise-time (ms)	0.14 ± 0.03	0.14 ± 0.03	0.17 ± 0.06	0.18 ± 0.07	0.138	0.744	0.052
AP half-height width (ms)	0.76 ± 0.13	0.64 ± 0.10	0.81 ± 0.38	0.77 ± 0.35	0.629	**0.002**	**0.041**
AP maximum rise ($mV.ms^{-1}$)	432.3 ± 113.1	460.7 ± 136.4	396.2 ± 130.4	354.5 ± 134.6	0.154	0.356	**0.015**
AP maximum decay ($mV.ms^{-1}$)	101.9 ± 16.3	139.8 ± 29.9	111.7 ± 42.5	112.1 ± 44.6	0.654	**0.0001**	**0.0004**
IF Slope ($AP. pA^{-1}$)	0.027 ± 0.020	0.042 ± 0.014	0.045 ± 0.030	0.044 ± 0.026	0.444	**0.004**	0.071

Supplementary Table 3: Comparison of electrophysiological properties of L2/3 and L5 PCs in adult rats and adult humans. Key intrinsic physiological measurements made from L2/3 and L5 PCs. Data is shown as mean ± SD. Statistics shown as p-values from linear mixed effects models (LMM) comparing the effect of species (spec.) age, and the interaction of both. Number of cells and rats contributing to each dataset are indicated.

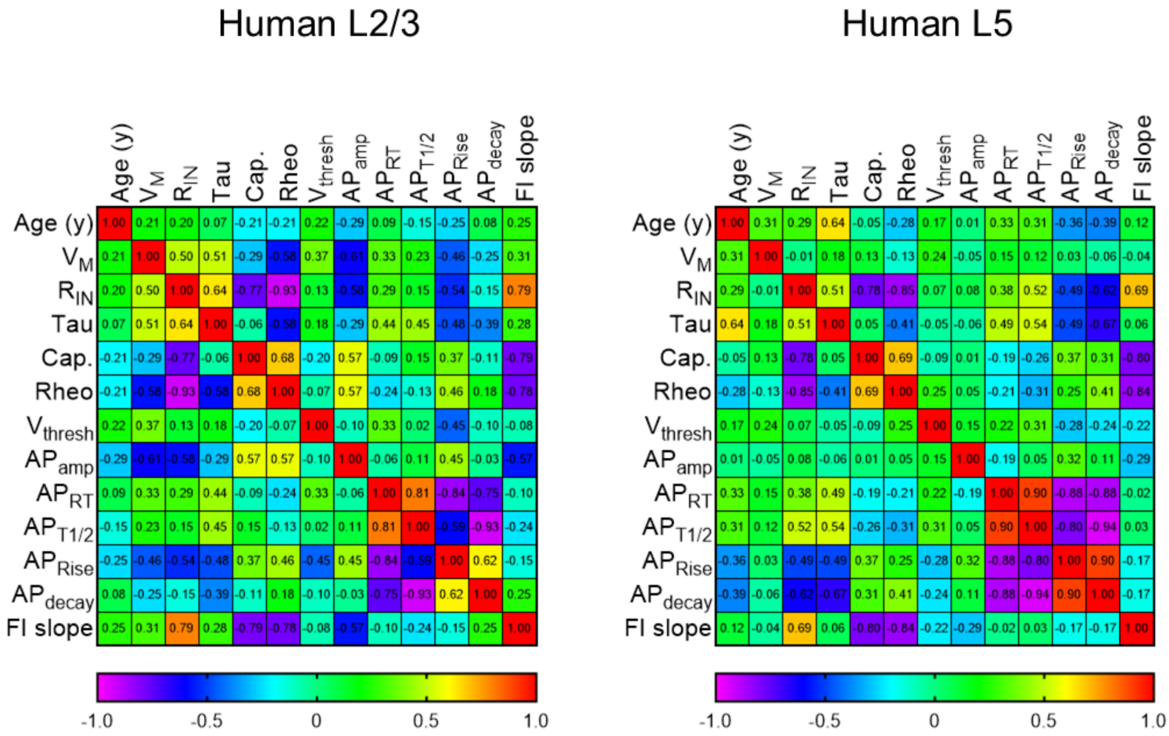

Supplementary Figure 1: Electrophysiological properties of human cortical pyramidal cells minimally correlates with age. Spearman correlation analysis of age (years) and key electrophysiological properties of identified human pyramidal neurons in L2/3 (left, 37 cells/20 patients) and L5 (right; 16 cells/9 patients). Note few cellular physiological properties closely correlated with age. Other key parameters show strong correlations in clusters, in particular passive membrane and AP properties.

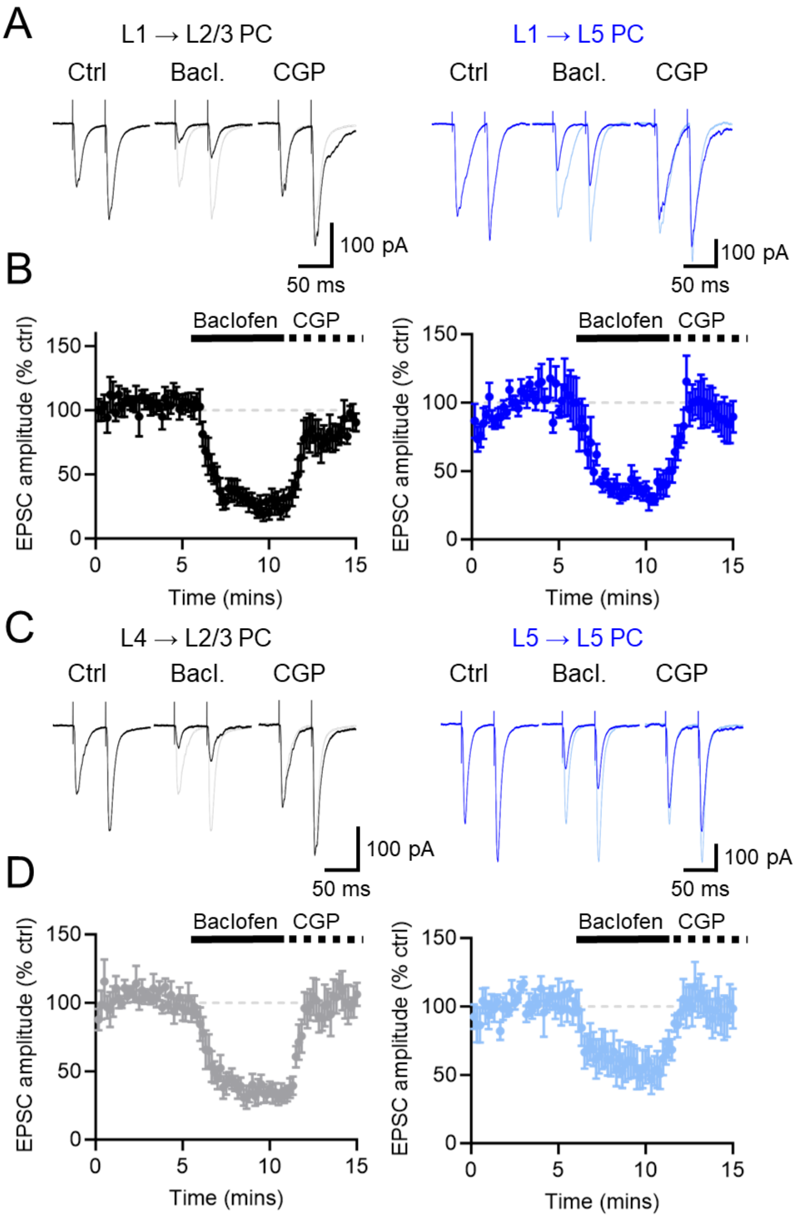

Supplementary Figure 2: Presynaptic GABA$_B$R-mediated inhibition of synaptic inputs to 6-8 month old rats. **(A)** Example EPSCs evoked by L1 stimulation in L2/3 PCs (6-8 month, 8 cells, 5 rats) recorded at -70 mV voltage clamp under control conditions (Ctrl) and following bath application of 10 µM baclofen (Bacl.) and 5 µM CGP-55,845 (CGP). Control recordings are shown for reference (grey traces). Lower, time-course of EPSC amplitude following baclofen (solid bar) and CGP (dashed bar) wash-in. Control baseline is shown for reference (grey dashed line). (**B**) Data in the same form but for L1 inputs to L5 PCs (blue; 9 cells, 5 rats). (**C**) Data in the same form, but for L4 inputs to L2/3 PCs (light grey). (**D**) Data in the same form, but for L5 inputs to L5 PCs (light blue). Data is shown as mean ± SEM.

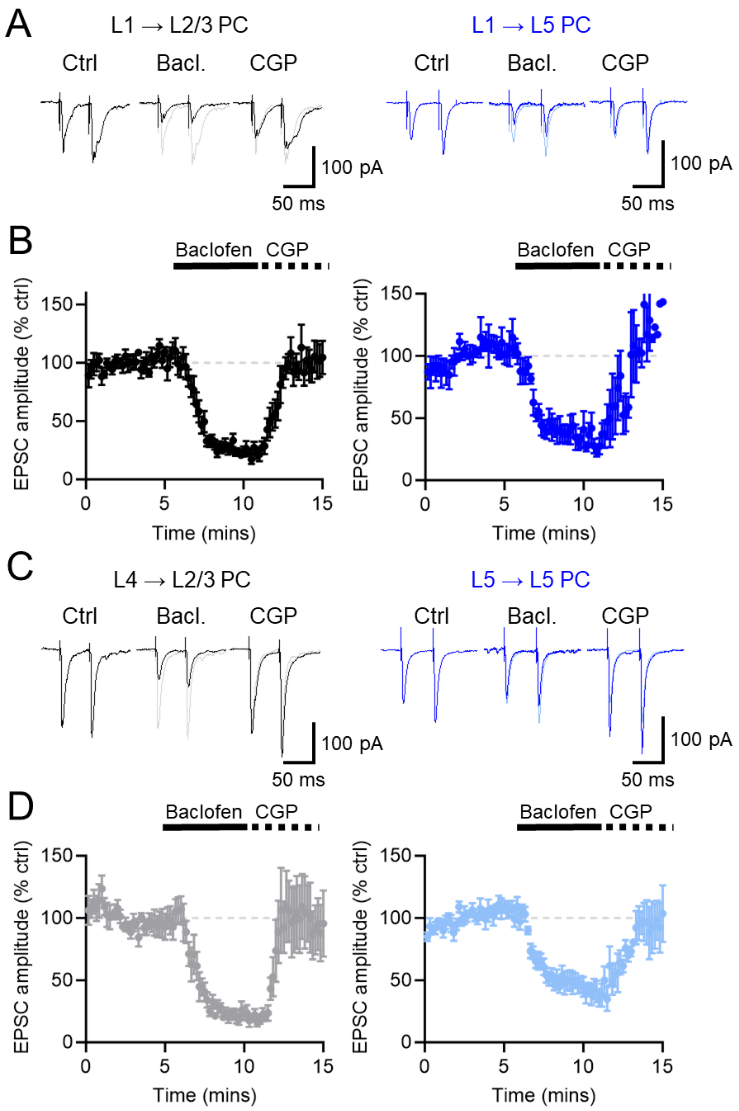

Supplementary Figure 3: Presynaptic GABA$_B$R-mediated inhibition of synaptic inputs to 1 month old rats. **(A)** Example EPSCs evoked by L1 stimulation in L2/3 PCs (1 month, 8 cells, 4 rats) recorded at -70 mV voltage clamp under control conditions (Ctrl) and following bath application of 10 µM baclofen (Bacl.) and 5 µM CGP-55,845 (CGP). Control recordings are shown for reference (grey traces). Lower, time-course of EPSC amplitude following baclofen (solid bar) and CGP (dashed bar) wash-in. Control baseline is shown for reference (grey dashed line). (**B**) Data in the same form but for L1 inputs to L5 PCs (blue; 8 cells, 4 rats). (**C**) Data in the same form, but for L4 inputs to L2/3 PCs (light grey). (**D**) Data in the same form, but for L5 inputs to L5 PCs (light blue). Data is shown as mean ± SEM.

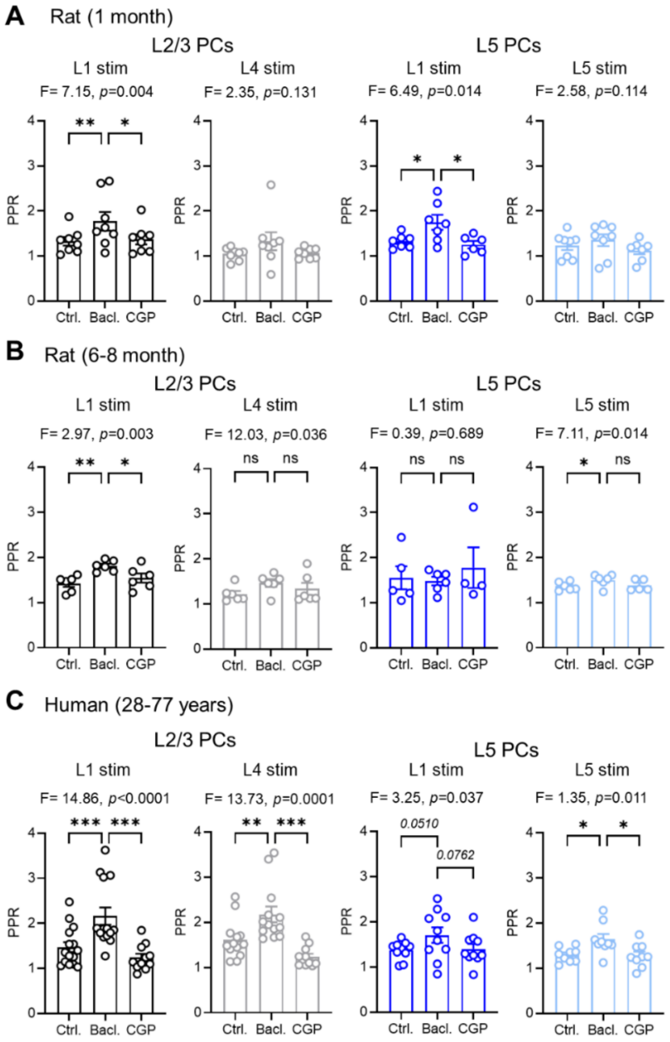

Supplementary Figure 4: Presynaptic short-term plasticity regulate by GABA$_B$Rs (A) PPR before (Ctrl) and after bath application of 10 µM baclofen (Bacl.) or 5 µM CGP-55,845 (CGP) at L1 (black) and L4 (grey) inputs to L2/3 PCs (n=8 cells/4 rats) and L1 (blue) and L5 (light blue) inputs to L5 PCs (n=7 cells/4 rats) in 1 month old rats (L2/3 [L1 stimulation]: Ctrl/Bacl. p= 0.006, Bacl./CGP p= 0.012, Ctrl/CGP p= 0.925; L5 [L1 stimulation]: Ctrl/Bacl. p= 0.031, Bacl./CGP p= 0.021, Ctrl/CGP p= 0.929; Tukey post-tests). (B) Data in the same form for 6-8 month-old rats (L2/3 8 cells/5 rats; L5 9 cells/5 rats; L2/3 [L1 stimulation]: Ctrl/Bacl. p= 0.003, Bacl./CGP p= 0.026, Ctrl/CGP p= 0.350; L5 [L5 stimulation]: Ctrl/Bacl. p= 0.014, Bacl./CGP p= 0.066, Ctrl/CGP p= 0.726; Tukey post-tests). (C) Data in the same form for 28-77-year-old human cortex (L2/3: 15 cells/7 cases; L5: 10 cells/3 cases). L2/3 [L1 stimulation]: Ctrl/Bacl. p= 0.0006, Bacl./CGP p= 0.0002, Ctrl/CGP p= 0.653; L2/3 [L4 stimulation]: Ctrl/Bacl. p= 0.007, Bacl./CGP p= 0.0001, Ctrl/CGP p= 0.134; L5 [L1 stimulation]: Ctrl/Bacl. p= 0.051, Bacl./CGP p= 0.076, Ctrl/CGP p= 0.977; L5 [L5 stimulation]: Ctrl/Bacl. p= 0.024, Bacl./CGP p= 0.018, Ctrl/CGP p= 0.990; Tukey post-tests). All data is shown as mean ± SEM (due to pairwise comparisons) and is shown with results from individual cells. Statistics shown: ns – p>0.05, * - *p*<0.05, ** - *p*<0.01 *** - *p*<0.001, all 1-way ANOVA with Tukey post-tests.

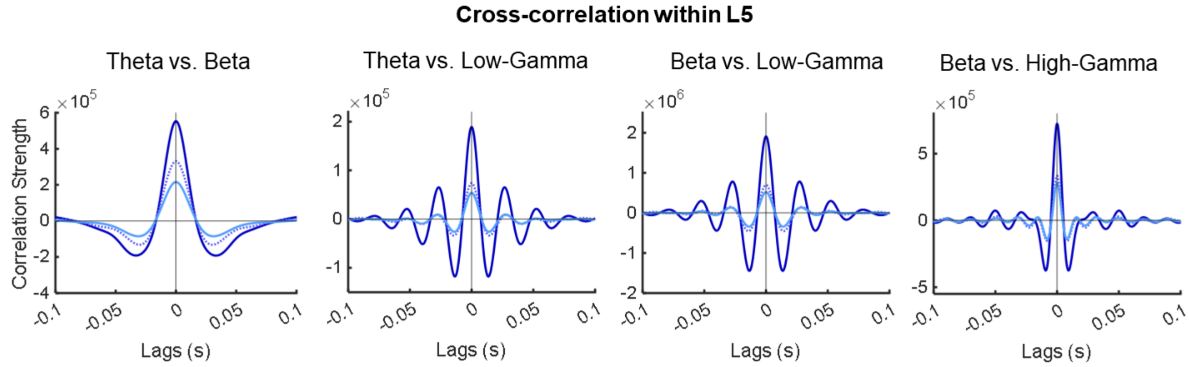

Supplementary Figure 5: Human L5 displays reduced correlation following baclofen bath application. Example plots of cross-correlation strength between prominent oscillations in LFP recordings from human L5 under control conditions (KA/CCh, blue) and following 2 µM (light blue) or 20 µM (lightest blue) baclofen bath application. Data is shown as example correlations.

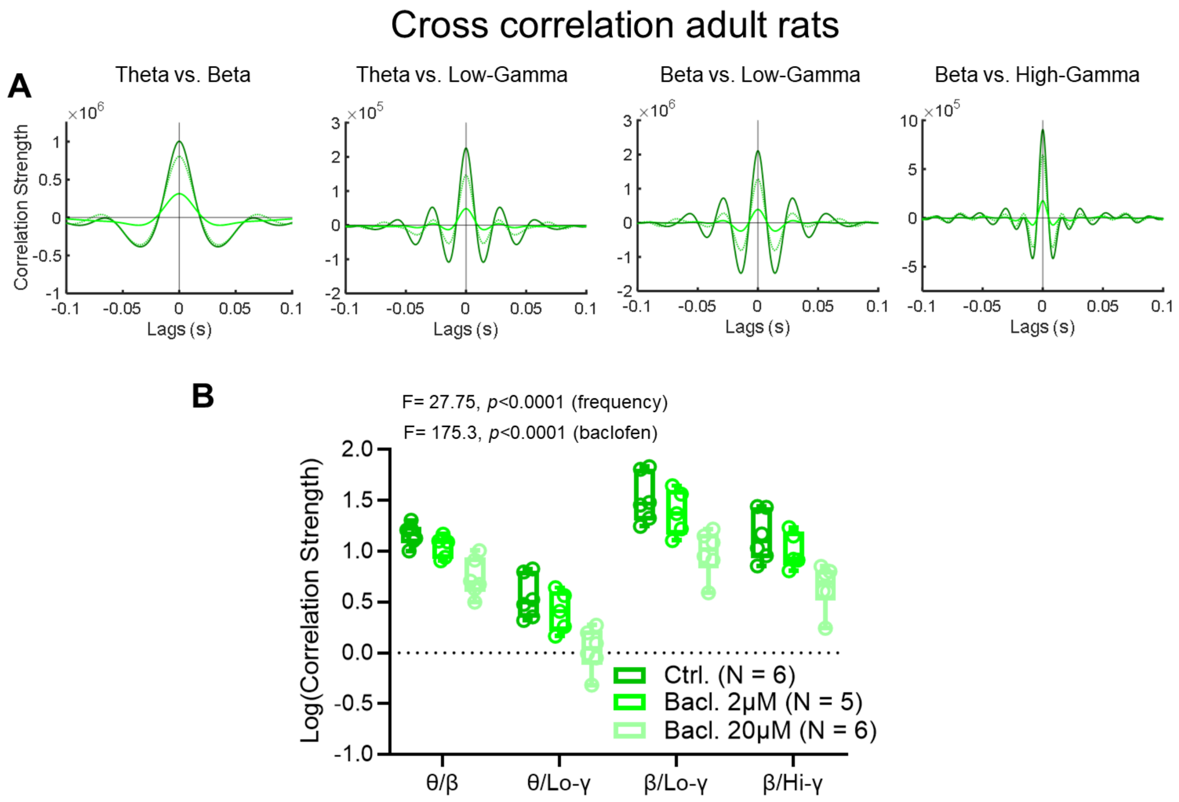

Supplementary Figure 6: Adult rat L2/3 PCs display reduced cross-correlation of oscillatory coupling following baclofen bath application. **(A)** Example plots of cross-correlation strength between prominent oscillations in LFP recordings from adult rat L2/3 from somatosensory cortex under control conditions (KA/CCh, green) and following 2 μM (light green) or 20 μM (lightest green) baclofen bath application. (**B**) Comparison of log-transformed correlation strength at zero lag (no time delay) between key oscillatory bands in L2/3 of the adult rat revealed a significant reduction in frequency due to baclofen from control (KA/CCh: n= 6 slices from 6 rats) in both 2 μM (5 slices from 5 rats) and 20 μM baclofen (6 slices from 6 rats) recordings. Data are shown as boxplots (25 - 75% range) with median indicated and whiskers indicating maximum/minimum ranges with individual data points overlain. Statistics are shown from 2-way ANOVA (2-sided) and results indicated on the graph.

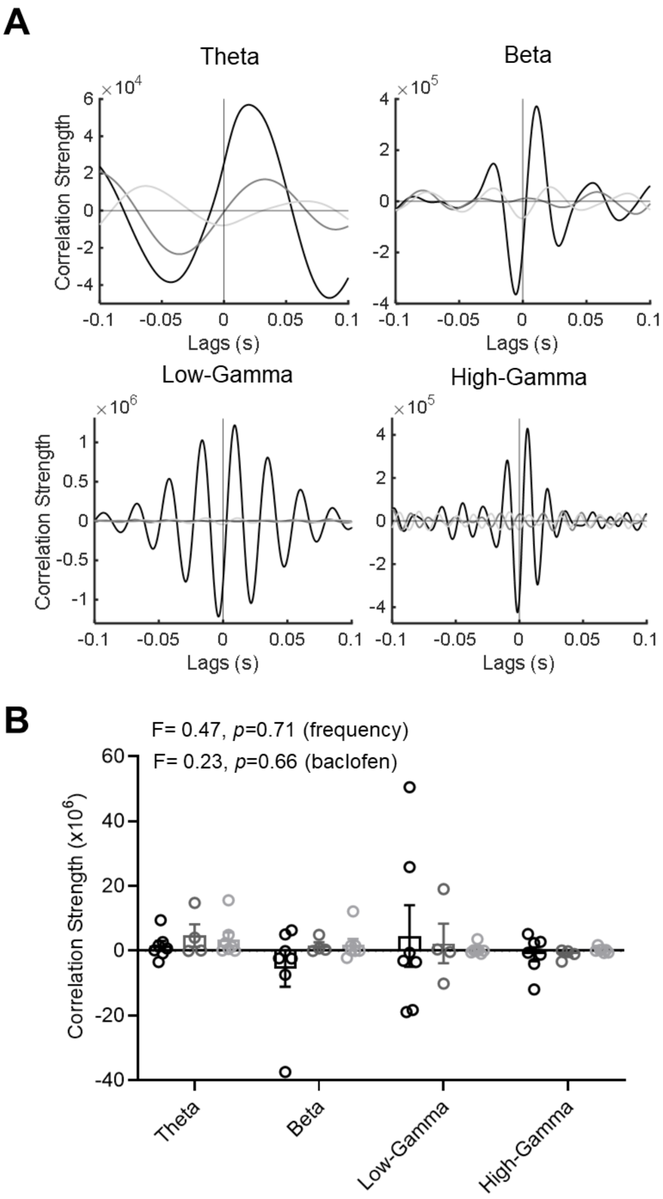

Supplementary Figure 7: Cross-correlation strength of prominent oscillations between cortical layers reveals minimal effect of baclofen in vitro. (**A**) Example cross-correlograms showing the strength of interaction between similar frequency oscillations under control conditions (KA+CCh, Black) and following 2 µM (grey) and 20 µM (light grey) baclofen application, with respect to lag time. (**B**) Grouped cross-correlation data showing positive and negative correlation across simultaneous oscillatory activity under control conditions (7 slices from 7 cases), and following 2 µM (grey, 4 slices from 4 cases) and 20 µM (light grey; 7 slices from 7 cases) baclofen application revealed no effect of baclofen. Data are shown as mean ± SEM (due to paired comparisons) with values from individual slices overlaid. Results from 2-way ANOVA (2-sided) are indicated on the graph.

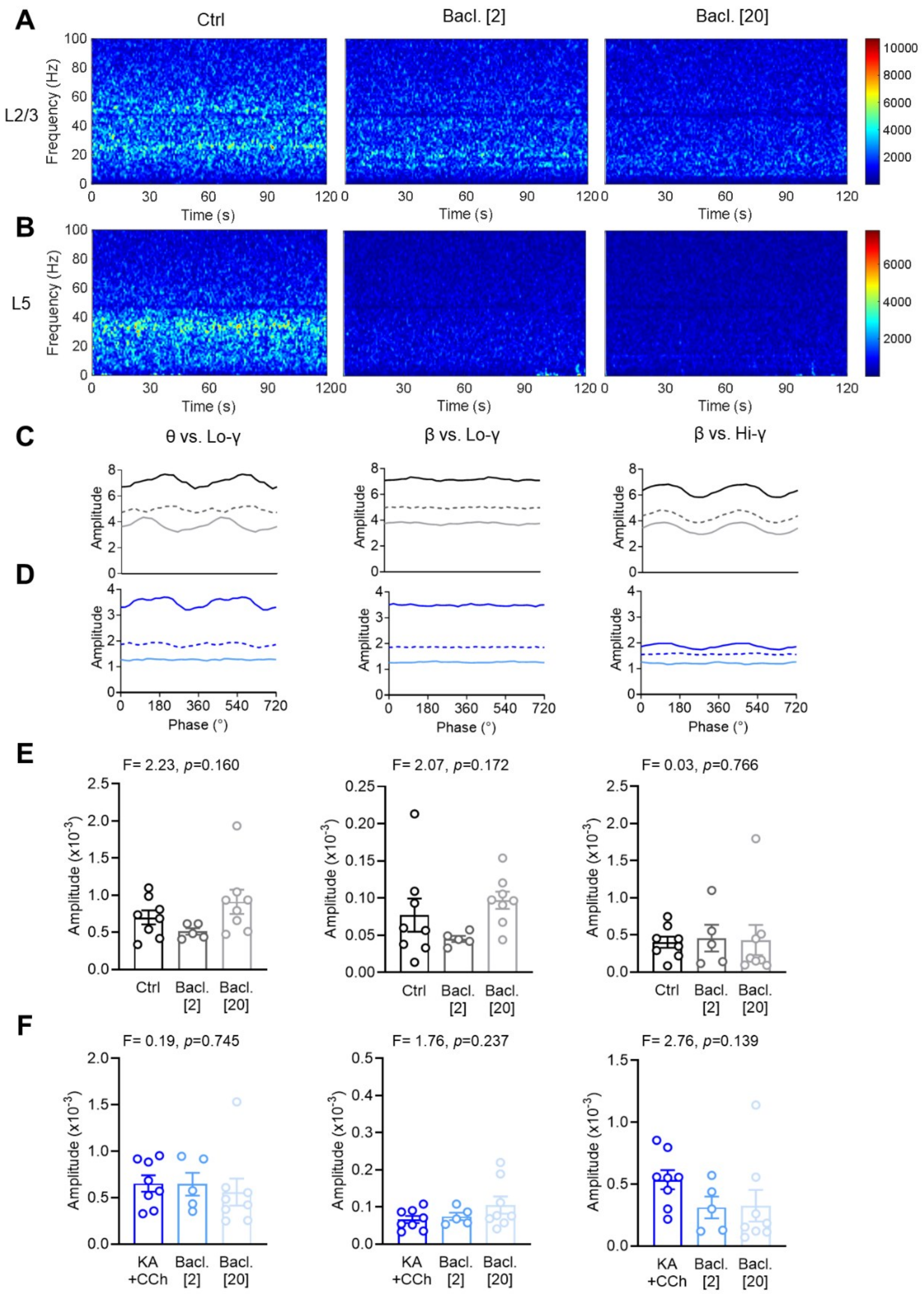

Supplementary Figure 8: Phase amplitude coupling in adult human brain slices reveals oscillatory coupling persists in the presence of baclofen. (**A**) Example phase-amplitude plots of 2 minute signal epochs of L2/3 under control (KA+CCh, left) and following 2 µM (middle) and 20 µM (right) baclofen application. Phase amplitude is shown as colour coded as low (blue) to high (red) amplitude coupling strength, and is unitless. (**B**) Similar representation as in **A**, but in L5 recordings. (**C**) Example phase-amplitude plots reflecting the strength (unitless)

between oscillations as a function of the phase of the lower frequency oscillation between prominent oscillations in L2/3 control conditions (solid, black) and after 2 μM (dashed, grey) and 20 μM (solid, light grey) baclofen application. (**D**) The same as **C**, but for L5 recordings under control conditions (solid, blue) and after 2 μM (dashed, blue) and 20 μM (solid, light blue). (**E**) Peak modulation index amplitudes between each frequency band in L2/3 under control (black; 8 slices from 8 cases) and following 2 μM (grey, 5 slices from 5 cases) and 20μM (light grey, 8 slices from 8 cases) baclofen application. (**F**) The same data, but for L5 - control (blue; 8 slices from 8 cases) and following 2 μM (mid blue, 5 slices from 5 cases) and 20μM (light blue, 8 slices from 8 cases) baclofen application. Data is shown as mean ± SEM (E, F). Statistics are reported from 1-way ANOVA above each graph.

Electrophysiological Property	Control (37 cells from 20 patients)	Seizures/LEV (34 cells from 15 patients)	LEV (13 cells from 4 patients)	LMM (group effect)		Ctrl vs. SZ/Lev	Ctrl vs. LEV	SZ/LEV vs. LEV
				F	P			
Membrane potential (mV)	-65.5 ± 5.9	-72.5 ± 6.3	-74.8 ± 4.2	4.61	**0.015**	0.177	**0.050**	0.559
Input resistance (MΩ)	120.0 ± 101.9	95.3 ± 64.1	66.2 ± 22.7	0.67	0.522			
Membrane time-constant (ms)	16.1 ± 7.9	15.1 ± 1.1	15.0 ± 3.8	0.14	0.869			
Capacitance (pF)	183.7 ± 86.6	202.7 ± 93.7	241.2 ± 69.9	1.36	0.274			
Rheobase (pA)	206.1 ± 136.3	258.1 ± 150.8	276.9 ± 92.7	1.81	0.183			
Voltage threshold (mV)	-41.2 ± 5.9	-42.0 ± 6.3	-44.8 ± 5.3	1.62	0.221			
AP amplitude (mV)	112.2 ± 10.5	116.1 ± 10.8	121.5 ± 6.1	2.58	0.105			
AP 20-80% rise-time (ms)	0.17 ± 0.06	0.17 ± 0.04	0.17 ± 0.06	0.15	0.860			
AP half-height width (ms)	0.81 ± 0.38	0.75 ± 0.19	0.80 ± 0.33	0.25	0.780			
AP maximum rise ($mV.ms^{-1}$)	396.2 ± 130.4	386.9 ± 121.3	456.5 ± 93.6	0.13	0.882			
AP maximum decay ($mV.ms^{-1}$)	111.7 ± 42.5	112.2 ± 24.6	109.8 ± 26.7	0.24	0.786			
IF Slope ($AP.\ pA^{-1}$)	0.045 ± 0.030	0.032 ± 0.023	0.031 ± 0.013	2.35	0.119			

Supplementary Table 4: Comparison of intrinsic electrophysiology parameters of L2/3 PCs recorded in control patients (no seizures or levetiracetam [LEV]), patients who had experienced seizures and been prescribed LEV, or patients who had not experienced seizures, but had been prescribed LEV. All data are shown as mean ± SD, from cell average data. Statistics are shown linear mixed-effects modelling with Tukey post-hoc tests when appropriate.
