# Supplementary material for: Phylogenetic divergence of GABAB receptor signaling in neocortical networks over adult life
Source: Nat Commun. 2025 May 6;16:4194. doi: 10.1038/s41467-025-59262-8 (PMC12056048; doi:10.1038/s41467-025-59262-8)
Supplement: Supplementary file 2 — Reporting Summary [file 41467_2025_59262_MOESM2_ESM.pdf]

nature portfolio

Corresponding author(s): Sam Booker

Last updated by author(s): Jan 24, 2025

Reporting Summary

Nature Portfolio wishes to improve the reproducibility of the work that we publish. This form provides structure for consistency and transparency in reporting. For further information on Nature Portfolio policies, see our Editorial Policies and the Editorial Policy Checklist.

Statistics

For all statistical analyses, confirm that the following items are present in the figure legend, table legend, main text, or Methods section.

n/a	Confirmed	
☐	☒	The exact sample size (*n*) for each experimental group/condition, given as a discrete number and unit of measurement
☐	☒	A statement on whether measurements were taken from distinct samples or whether the same sample was measured repeatedly
☐	☒	The statistical test(s) used AND whether they are one- or two-sided<br>*Only common tests should be described solely by name; describe more complex techniques in the Methods section.*
☐	☒	A description of all covariates tested
☐	☒	A description of any assumptions or corrections, such as tests of normality and adjustment for multiple comparisons
☐	☒	A full description of the statistical parameters including central tendency (e.g. means) or other basic estimates (e.g. regression coefficient) AND variation (e.g. standard deviation) or associated estimates of uncertainty (e.g. confidence intervals)
☐	☒	For null hypothesis testing, the test statistic (e.g. *F*, *t*, *r*) with confidence intervals, effect sizes, degrees of freedom and *P* value noted<br>*Give P values as exact values whenever suitable.*
☒	☐	For Bayesian analysis, information on the choice of priors and Markov chain Monte Carlo settings
☒	☐	For hierarchical and complex designs, identification of the appropriate level for tests and full reporting of outcomes
☐	☒	Estimates of effect sizes (e.g. Cohen's *d*, Pearson's *r*), indicating how they were calculated

Our web collection on statistics for biologists contains articles on many of the points above.

Software and code

Policy information about availability of computer code

Data collection	All data was collected with proprietary software (pClamp, Spike2, Leica LASX).
Data analysis	All data was analysed using either Stimfit , Spike 2, ImageJ, or MATLAB scripts (see supplementary materials).

For manuscripts utilizing custom algorithms or software that are central to the research but not yet described in published literature, software must be made available to editors and reviewers. We strongly encourage code deposition in a community repository (e.g. GitHub). See the Nature Portfolio guidelines for submitting code & software for further information.

Data

Policy information about availability of data

All manuscripts must include a data availability statement. This statement should provide the following information, where applicable:

- Accession codes, unique identifiers, or web links for publicly available datasets
- A description of any restrictions on data availability
- For clinical datasets or third party data, please ensure that the statement adheres to our policy

Source data contributing to figures is submitted as supplementary materials - further data available upon request

Research involving human participants, their data, or biological material

Policy information about studies with human participants or human data. See also policy information about sex, gender (identity/presentation), and sexual orientation and race, ethnicity and racism.

Reporting on sex and gender	All data pertaining to biological sex of patients from whom tissue was collected has been provided in supplementary materials. All permissions to utilise such data are in line with Caldicott approvals - as stated in the methods. No analyses examined sex-differences - despite both sexes being sampled.
Reporting on race, ethnicity, or other socially relevant groupings	No variables relating to race, ethnicity or socially relevant grouping are provided in this manuscript.
Population characteristics	specific data is provided for 2 variables - biological sex, and treatment profile for all individuals utilised in this study. These data are provided in Supplementary Table 1.aggregate age and clinical diagnosis is shown to avoid inadvertent identification of patients.
Recruitment	Patients were consented to give neurosurgical access tissue as part of the normal consenting procedure. All patients were recruited to the study by an independent research nurse team. Given the nature of the study (comparing neuronal signalling between different patients and the wide range of ages and clinical diagnoses) there is likely little to no impact on the results. Indeed, we highlight differences between these cohorts
Ethics oversight	Ethical approval was provided by: UK NHS Lothian: REC number: 15/ES/0094, IRAS number: 165488; and UK NHS Newcastle: IRAS 173990

Note that full information on the approval of the study protocol must also be provided in the manuscript.

Field-specific reporting

Please select the one below that is the best fit for your research. If you are not sure, read the appropriate sections before making your selection.

☒ Life sciences ☐ Behavioural & social sciences ☐ Ecological, evolutionary & environmental sciences

For a reference copy of the document with all sections, see nature.com/documents/nr-reporting-summary-flat.pdf

Life sciences study design

All studies must disclose on these points even when the disclosure is negative.

Sample size	Sample size was based on our previous publications characterisating GABAB receptor signalling, where greatest variability exists between cells, and less so between biological replicates As such, we aimed to gather a minimum of 5 biologiical replicates per experimental condition. For 1 data set (patients recieving Levetiracetam but not experiencing seizures), this was hampered by this being a rare clinical group which we only gather 4 biological replicates.
Data exclusions	No data were excluded.
Replication	A key finding (the elevated GABAB receptor signalling in patients receiving Levetiracetam) was repeated on a separate cohort (n=4 patients), which confirmed the original finding. This data is shown in Figure 8F
Randomization	All experiments were performed in either wild-type rats or human brain samples. Given the nature of surgical sample availability, there was no choice about which sample was obtained.
Blinding	For majority of patients experiencing seizures, we did not have clinical information a priori - thus we were blind to clinical history.

Reporting for specific materials, systems and methods

We require information from authors about some types of materials, experimental systems and methods used in many studies. Here, indicate whether each material, system or method listed is relevant to your study. If you are not sure if a list item applies to your research, read the appropriate section before selecting a response.

Materials & experimental systems

n/a	Involved in the study
☐	☒ Antibodies
☒	☐ Eukaryotic cell lines
☒	☐ Palaeontology and archaeology
☐	☒ Animals and other organisms
☒	☐ Clinical data
☒	☐ Dual use research of concern
☒	☐ Plants

Methods

n/a	Involved in the study
☒	☐ ChIP-seq
☒	☐ Flow cytometry
☒	☐ MRI-based neuroimaging

Antibodies

Antibodies used	GABAB1 (Neuromab, N93A_49; RRID: AB_10672843)
Validation	This antibody has been previously validated in wild-type and knock-out tissue by the manufacturers and is openly available for download (https://neuromab.ucdavis.edu/datasheet/N93A_49.pdf)

Animals and other research organisms

Policy information about studies involving animals; ARRIVE guidelines recommended for reporting animal research, and Sex and Gender in Research

Laboratory animals	This study involved Long Evans-Hooded rats (male and female). Rats were used at 1 month (28-35 days) or 6-8 months or 12-14 months of age
Wild animals	n/a
Reporting on sex	Male and Female rats and human samples were used for all experiments. Based on our statistical modelling, where sufficient sex-specific replicates were included, no variability was observed between sex. Our data wanted to sample from the human population as a whole, as this has greatest clinical relevance, as such sex was not defined as a fixed effect for analysis.
Field-collected samples	n/a
Ethics oversight	All rodent experiments were performed in line with the UK Home Office (ASPA 1986) guidelines, under UK HO Project license PP2262639. All procedures were performed in accordance with University of Edinburgh Guidelines.

Note that full information on the approval of the study protocol must also be provided in the manuscript.

Plants

Seed stocks	n/a
Novel plant genotypes	n/a
Authentication	n/a
